# Isolated radial volar dislocation of the fifth carpometacarpal joint :a rare injury

**DOI:** 10.11604/pamj.2013.16.90.3218

**Published:** 2013-11-10

**Authors:** Khalid Ibn El Kadi, Mohcine Sbiyaa, Badr Alami, Ilyass Rabhi, Amine Marzouki, Kamal Lahrach, Fawzi Boutayeb

**Affiliations:** 1 Department of Orthopedic Surgery (A), UH Hassan II,Fes, Morocco

**Keywords:** Volar dislocation, carpometacarpal, diagnosis

## Abstract

Isolated palmar dislocation of the fifth carpometacarpal joint is an uncommon injury and classified as radio-palmar or ulno-palmar according to the direction of displacement of the fifth metacarpal base. This very rare injury is often difficult to recognize. A careful neurologic assessment of the patient is a necessity, as well as obtaining proper radiographs of the hand. The purpose of this report is to present a patient with a pure isolated volar dislocation of the fifth carpometacarpal joint that was satisfactorily treated with closed reduction and casting. A review of the literature is presented.

## Introduction

Volar dislocation of the fifth carpometacarpal joint is an uncommon injury. Only few cases previously had been described in the consulted English literature [1–4]. We report a case of volar dislocation of the base of fifth carpometacarpal joint satisfactorily treated with closed reduction and casting. The mechanism of this injury, clinical presentation and treatment options are discussed, with a review of the literature.

## Patient and observation

**Figure 1:**
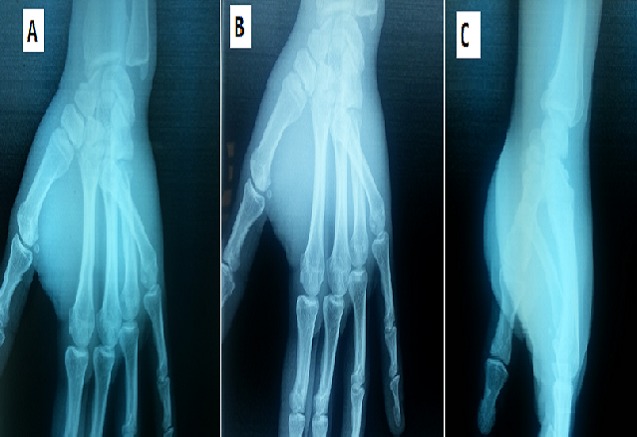
Anteroposterior (A) ,oblique (B) and lateral(C) X-rays showing radiopalmar dislocation of the fifth carpometacarpal joint

A 25-year-old man complained of severe pain on his right hand after a fall when he suffered a direct traumatism over ulnar-dorsal side of the hand. Physical examination showed localised tenderness and moderate swelling over the fifth metacarpal and along the ulnar side of the hand. The little finger presented a marked deformity with mild abduction and external rotation. There was a palpable prominence in the volar hypothenar region. Subjectively, sensation was intact in the median and ulnar nerve distributions. Also, vascular exploration of the hand was normal. Standard plain radiographs revealed a palmar and radial dislocation of the fifth carpometacarpal joint without other lesions or fractures on the others digits or the wrist (Figure 1).

**Figure 2:**
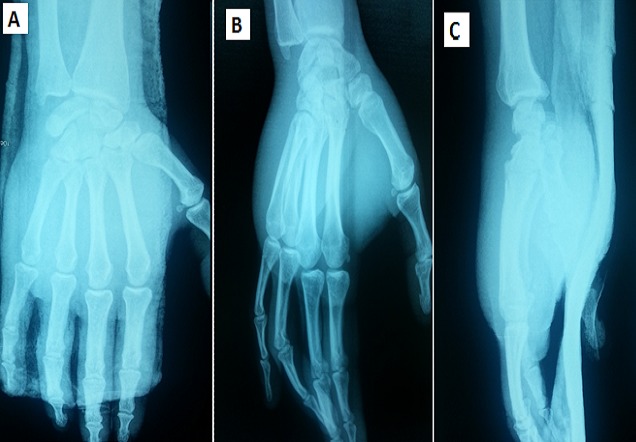
Post reduction of 5th CMC-joint : Anteroposterior view (A), oblique view (B) and lateral (C) view

Immediately, under local anaesthesia, we performed a closed reduction by longitudinal traction and direct pressure over volar base of the metacarpal. The reduction was stable and was confirmed by X-rays control (Figure 2).

**Figure 3:**
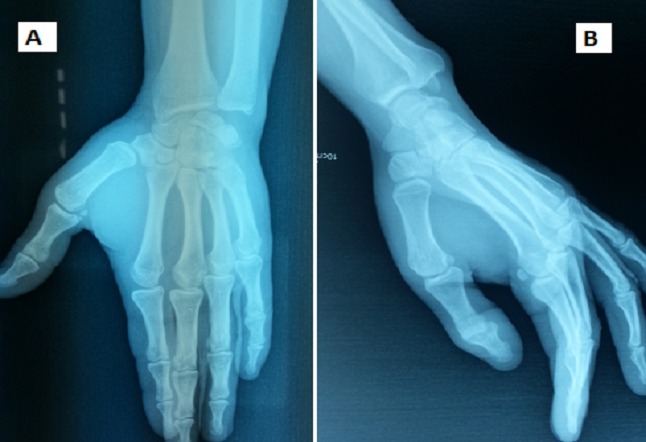
6-month follow-up: Reduction maintained. (A) AP view and (B) pronation view

After the wrist and the metacarpophalangeal joint of the little finger were immobilized in a cast for 6weeks. After this period of immobilization, the patient had been working with physical therapy and using his hand for daily activities. Six months following injury the patient had full range of movement of the little finger and normal grip strength, the radiographs showed the reduction to be maintained (Figure 3).

## Discussion

The normal fifth carpometacarpal joint is supported by a sturdy pisometacarpal ligament, dorsal and palmar carpometacarpal ligaments, and an intermetacarpal ligament [5, 6]. The intermetacarpal ligament reportedly constitutes the primary restraint. Also, the extensor carpi ulnaris and the flexor carpi ulnaris tendons insert into the base of the fifth metacarpal.

Carpometacarpal (CMC) joint dislocations are relatively uncommon injury and they occur in less than 1% of hand injuries [7]. Carpometacarpal joint dislocation is classified as dorsal and volar (so named palmar too) dislocations, and they may be associated to fractures of adjacent metacarpal o carpal bones. Dorsal dislocations of the carpometacarpal joints occur more frequent than volar, mainly affect to fourth and fifth fingers [8]. Dislocations of the fifth carpometacarpal joint associated with hamate fractures [9–11] or fractures of the base of the fifth metacarpal or with another metacarpal joint dislocations types are more frequent than isolated dislocations [4, 12–14]. Divergent simultaneous carpometacarpal joint dislocation involving differents digits is another rare type of lesion described by some authors [15]. Saleemi [16] reported a variety of these injuries and he described an isolated unilateral fifth CMC joint dislocation to the ulnar side, only seen in the postero-anterior (PA) view of hand X-ray.

Isolated volar or palmar dislocation of the fifth carpometacarpal joint is an uncommon injury that was first reported in 1918 by McWhorter [12]. In 1965 Nalebuff [6] classified the volar dislocations into two groups according to the direction of the displacement of the fifth metacarpal base: radial palmar and ulnopalmar. At the first one the fifth metacarpal base is completely denuded of any ligaments or tendon attachments. At the second type the pisometacarpal ligament and tendon attachments are intact [6, 7].

Although, it is more difficult for the patients to remember the exact traumatism suffered, the mechanism of this injury have been seemed a direct blow transmitted to the dorsal and ulnar aspect of the base of the fifth metacarpal. This injury caused a rupture of all ligaments and tendon attachments of the base of the fifth metacarpal [7].

The physical findings in this dislocation are pain and swelling about the base of fifth metacarpal and axial deformity of the little finger. It is possible to observe an apparent shortening of the affected metacarpal.

A careful neurologic evaluation must be performed. The deep motor branch of the ulnar nerve lies volar to the fifth CMC joint as it courses around the hook of the hamate. It is vulnerable to injury in both dorsal [17, 18] and volar [19] CMC dislocations.

Even though the simple radiology findings are usually enough to diagnose these lesions in the ulnar dislocations several authors reported that the radiograph diagnosis is often difficult and these lesions may be overlooked [10, 20]. Careful attention to the parallel lines of the carpometacarpal articular surfaces can help avoid this pitfall. The key of a correct X-ray diagnosis is a 30° pronated lateral view in which the fifth carpometacarpal joint is projected in profile to demonstrate the displacement [21]. Yamakado [20] described a case diagnosed correctly by simple stress X-rays (traction and axial compression stress views). Like own case, in radial palmar dislocation the X-ray diagnosis is easier due to the several displacement.

The treatment of ulnopalmar dislocation has evolved along the years: in previous publications none surgical treatment was applied with unsuccessful results [13]. Later, the close reduction and fixation with two K-wires before [4, 16], and only with one after [14], demonstrated good results. Finally, in 1986, Berg reported [1] the first successful case treated with none surgical methods: close reduction and maintenance of the reduction with a plaster. Since there, this treatment method was applied with successful results in other cases [22].

Typically, one would assume that this injury pattern would need some form of surgical intervention, as is the case with most radial volar CMC dislocations. We believe that timely diagnosis, adequate sedation with muscle relaxation, followed by gentle reduction maneuvers allowed us to achieve near-anatomic and stable reduction of the fracture dislocation, which was adequately treated conservatively. Steinberg [23] has published in 2013 a case of Acute closed radial dislocation of the second through fourth carpometacarpal joints treated conservatively with good results. Our patient demonstrated excellent functional results at 6-month follow-up and the anatomical reduction was maintained.

Remember that in this type of dislocation, all ligaments and tendons attachments of the base of fifth metacarpal results tearing and that might explain the instability which required surgical treatment by percutaneous pinning or open reduction and internal fixation^7^. However, as demonstrated in our case, correct diagnosis during the acute setting and timely intervention can potentially keep these patients out of the operating room. If this approach is chosen, close follow-up and serial radiographic evaluation is recommended to ensure maintenance of reduction and recovery of hand function.

## Conclusion

In conclusion, isolated radial palmar dislocation of the fifth carpometacarpal joint is an uncommon lesion. His intrinsic instability required surgical treatment. Conservative treatment is indicated if the closed reduction is stable.
